# Enhanced biological control of root-knot nematode, *Meloidogyne incognita*, by combined inoculation of cotton or soybean seeds with a plant growth-promoting rhizobacterium and pectin-rich orange peel

**DOI:** 10.21307/jofnem-2021-058

**Published:** 2021-06-22

**Authors:** Mohammad K. Hassan, Kathy S. Lawrence, Edward J. Sikora, Mark R. Liles, Joseph W. Kloepper

**Affiliations:** 1 Department of Entomology and Plant Pathology, Auburn University, Auburn, AL, 36849; 2 Department of Biological Sciences, Auburn University, Auburn, AL, 36849

**Keywords:** Biological control, *Meloidogyne incognita*, Orange peel, Pectin, Root-knot nematode

## Abstract

LC-MS analysis of plant growth-promoting rhizobacterium (PGPR) *Bacillus velezensis* AP203 supernatants indicated the presence of nematode-inhibiting compounds that increased in abundance when *B. velezensis* AP203 was grown on orange peel. *Meloidogyne incognita* J2 were incubated with *B. velezensis* AP203 spores and orange peel, spores alone, orange peel alone, or with a non-inoculated control, and the combination of *B. velezensis* AP203 with orange peel resulted in 94% mortality of *M. incognita* juveniles (*p* ≤ 0.05). The J2 mortality rate for *B. velezensis* alone was 53%, compared to 59% mortality with orange peel, and the non-inoculated control exhibited 7% mortality. When tested on soybeans raised in a greenhouse, it was observed that when grown in the presence of orange peel, *B. velezensis* AP203 culture broth, cell suspension or supernatant reduced the numbers of *M. incognita* eggs per g of root at 45 days after planting (DAP) compared to inoculated controls in soybean and cotton (*p* ≤ 0.05). Likewise, soybean root length and fresh root weight significantly increased after inoculation with *B. velezensis* AP203 amended with orange peel. In cotton, shoot and root length significantly increased after inoculation with cell pellets of *B. velezensis* AP203 amended with orange peel compared to the *M. incognita* inoculated control. These data indicate that *B. velezensis* AP203 responds to growth on pectin-rich orange peel by production of biologically active secondary metabolites that can promote plant growth and inhibit root-knot nematode viability.

Cotton (*Gossypium hirsutum* L.) and soybean (*Glycine max* L.) are economically important crops in the United States and worldwide. In the U.S. alone, cotton yield in 2018 was 18.4 million bales, and soybean yield was 4.54 billion bushels (Anonymous, 2018). *Meloidogyne incognita* (Kofoid and White) Chitwood, the southern root-knot nematode, is broadly distributed in soils cultivated with cotton (Xiang et al., 2017b) and other crops (Huang et al., 2016), and causes economically significant yield losses annually; for example, in 2018, soybean yield losses due to *M. incognita* in the southern U.S. were estimated at 11.92 million bushels in total, with 70,000 bushels lost in Alabama (Allen et al., 2018).

Multiple methods are used to reduce *M. incognita* populations in the field, including chemical nematicides (Basyony and Abo-Zaid, 2018); however, environmental and health concerns have limited the use of chemical nematicides for controlling plant-parasitic nematodes, and there is a need to develop environmentally friendly methods to manage the pathogen, such as the use of biological control agents (Burkett-Cadena et al., 2008).

Plant growth-promoting rhizobacteria (PGPR) are root-colonizing bacteria that enhance plant growth and biological control against multiple plant pathogens (Olanrewaju et al., 2017). *Bacillus velezensis* is a Gram-positive, rod-shaped PGPR, with some strains reported to reduce *M. incognita* populations on cotton (Xiang et al., 2017b). Additionally, *B. velezensis* PGPR strains are able to utilize pectin as a sole carbon and energy source (Hossain et al., 2015); interestingly, citrus peels which are pectin-rich and an abundant and inexpensive agricultural waste product (Rafiq et al., 2018) have been previously demonstrated to increase the efficacy of soybean growth promotion mediated by *B. velezensis* PGPR strains when co-inoculated with orange peel powder as a seed treatment (Hassan et al., 2019).

In the presence of different carbohydrates (pectin, sucrose, xylan or galactose), *Bacillus* spp. produce multiple secondary metabolites that inhibit the growth of multiple plant pathogens. For example, *Bacillus amyloliquefaciens* SQY 162 grown on pectin-amended media increased surfactin production and inhibit bacterial wilt of tobacco caused by *Ralstonia solanacearum* (Wu et al., 2015). Also supernatants, cell pellet suspensions, and culture broths of *B. subtilis* strains significantly reduced populations of eggs and J2 of *M. incognita* under laboratory and greenhouse conditions (Basyony and Abo-Zaid, 2018). In addition, previous studies have reported that the combination of separated cow manure and orange peels (SCM-OP) reduced the number of *Meloidogyne javanica* populations on tomato roots (Raviv et al., 2005). However, the effect of exogenous orange peel amendments has not been evaluated for enhanced PGPR-mediated biological control of plant pathogenic nematodes such as *M. incognita*.

The overall goal of this study was to evaluate the combined application of *B. velezensis* AP203 and orange peel for biological control of *M. incognita*. More specifically, we investigated (i) the effects of *B. velezensis* AP203, with and without orange peel amendment, on the mortality of second-stage juveniles (J2) of *M. incognita* under laboratory conditions; (ii) the effect of orange peel or glucose by *B. velezensis* AP203 on the expression of secondary metabolite(s) predicted to be responsible for reducing *M. incognita* populations; (iii) evaluate the efficacy of orange peel and/or *B. velezensis* AP203 amendments in reducing the number of *M. incognita* populations on the roots of soybean and cotton under greenhouse conditions.

## Materials and methods

### Evaluation of nematode killing under laboratory conditions

#### Preparation of *B. velezensis* spore and orange-peel suspensions

*B. velezensis* AP203 was obtained from the Biological Control lab in the Department of Entomology and Plant Pathology, Auburn University, Alabama, USA (retired director Prof. Joseph Kloepper). This strain was originally isolated from a cotton rhizosphere. The cryopreserved culture was streaked onto tryptic soy agar (TSA) and incubated at 28°C for 24 hr. A single colony of the strain was transferred into a spore preparation medium (Zhang et al., 2010), and incubated for seven days at 28°C. Sterilized distilled water (20 mL) was added to each petri plate, and the bacterial mass was transferred to a 50 mL centrifuge tube. The *B. velezensis* AP203 spore preparation was heat-treated for 20 min at 80°C to kill any vegetative cells, serially diluted, and adjusted to 10^7^ colony forming units (CFU) per mL. The non-organic orange peel powder (Citrus Extracts LLC, Fort Pierce, FL USA) was suspended in sterilized distilled water by a magnetic stirrer at a rate of 1.0 g per 100 mL (1.0% w/v) water and was applied as an aqueous suspension.

#### Preparation of *M. incognita* inoculum and mortality determination

*M. incognita* eggs were isolated and extracted from corn plant roots Mycogen 2H273 (Dow AgroScience, Indianapolis, IN) at the Plant Science Research Center (Auburn University, Auburn, AL) using a modified sucrose centrifugation-flotation method (Jenkins, 1964). The eggs were counted using an inverted TS100 Nikon microscope at 40X magnification. The eggs were hatched for a seven-day period at 30°C in an incubator. In total, 10 µL of J2 *M. incognita* were counted and transferred into a 96-well plate for the J2 mortality test, with 30 to 40 J2 per well. In total, 90 µL of AP203 alone, citrus extract alone, and AP203 with citrus extract combined were added to each well. The 96-well plate was sealed with parafilm and incubated at room temperature for 48 hr. The number of live J2 were counted at the beginning (0 hr) and at the end (48 hr) of this experiment. The viability of J2 was determined by the sodium hydroxide method (Xiang and Lawrence, 2016) and the mortality percentage was calculated by the equation: [(live J2 at 0 hr‒live J2 at 48 hr)/live J2 at 0 hr] × 100 (Xiang et al., 2017b).

### LC-MS experiment

#### Preparation of *B. velezensis* AP203

*B. velezensis* AP203 was prepared as previously described. A single colony of the PGPR strain was transferred into TSA, Tris-Spizizen Salts medium (TSS), or TSS + orange peel powder (OPP; 0.5% w/v) media and grown for 72 hr in a shaking incubator at 28°C. The *B. velezensis* AP203 cultures were subjected to centrifugation in a Sorvall Legend RT centrifuge (Thermo Scientific, USA) at 10,000 × *g* for 10 min. The supernatant was collected and passed through a 0.2 µm syringe filter (VWR, Radnor, PA, USA) and was then transferred into a 1 mL microcentrifuge tube for LC-MS analysis.

#### LC-MS analysis

LC-MS analysis was performed at the Auburn University Mass Spectrometry Center using an ultra-performance LC system (ACQUITY, Waters Corp., USA) coupled with a quadrupole time-of-flight mass spectrometer (Q-Tof Premier, Waters) with electrospray ionization (ESI) in positive and negative mode using Masslynx software (V4.1). A 10 µl sample was injected into a C4 column (Aeris Widepore C4, 3.6 µm, 2.1 × 50 mm, Phenomenex) with a 300 μ L/min flow rate of the mobile phase. In positive mode, the mobile phase was solution A (0.1% formic acid in water) and solution B (95% acetonitrile, 5% H_2_O, and 0.1% formic acid) was initiated beginning at 0% B, held for 2 min, then linear ramping to 50% B in 18 min, followed by ramping to 100% B in 8 min and held at 100% B for 2.5 min, and then back to 0% B in 0.5 min with 4 min of re-equilibration at 0% B. In negative mode, the mobile phase was solution A (2 mM ammonium formate in water) and solution B (100% acetonitrile) beginning at 2% B, held for 2 min, then linear ramping to 50% B in 18 min, followed by ramp to 95% B in 8 min, held at 95% B for 2.5 min, and back to 2% B in 0.5 min with 4 min of re-equilibration at 2% B. The capillary voltage was set at 3.1 kV in positive mode and 2.8 kV in negative mode, the sample cone voltage was 30 V, and the extraction cone was 4.3 V. The source and desolvation temperature were maintained at 105 and 300 °C, respectively, with the desolvation gas flow at 600 L/hr. The Time of Flight Mass Spectrometry (TOF/MS) scan was 1 sec long from 80 to 1400 m/z with a 0.02 sec inter-scan delay using the centroid data format. The lock mass was used to correct instrument accuracy with a 2.5 µg/mL solution of leucine encephalin (Bachem H-2740). The data were converted to mzXML and analyzed with XCMS Online (Huan et al., 2017).

#### Greenhouse experiment

The greenhouse test was to evaluate the combined effects of *B. velezensis* AP203 plus orange peel or glucose for biological control of *M. incognita*. The treatment groups for the greenhouse study included: (i) TSB-CELLS + *M. incognita*, (ii) SUPERNATANT-OPP + *M. incognita*, (iii) SUPERNATANT-Glucose + *M. incognita*, (iv) TSS-CELLS-OPP + *M. incognita*, (v) TSS-CELLS-Glucose + *M. incognita*, (vi) CULTURE-OPP + *M. incognita*, (vii) CULTURE-Glucose + *M. incognita*, (viii) TSS + OPP (no bacteria present) + *M. incognita*, or (ix) TSS + glucose (No bacteria present) + *M. incognita,* (x) a positive control with *M. incognita*, and (xi) a negative control without *M. incognita*.

#### Preparation of *B. velezensis* AP203 and orange-peel suspension

*B. velezensis* AP203 was grown from a cryostock onto Tryptic Soy Agar (TSA) at 28°C for 24 hr. A single colony of this strain was transferred into TSA, TSS + glucose (0.5% w/v), or TSS + OPP (0.5% w/v) media and grown for 48 hr in a shaking incubator at 28°C. The *B. velezensis* cultures were then subjected to centrifugation in a Sorvall Legend RT centrifuge (Thermo Scientific, USA) at 10,000 × *g* for 10 min and each was then adjusted to approximately10^7^ CFU/mL based on culture turbidity at an optical density of 600 nm (OD_600_). The TSA grown strain was suspended in TSS broth based on OD_600_ and an aliquot (1 mL containing ~10^7^ CFU) of this sample (TSA-CELLS) was applied to each seed. For *B. velezensis* AP203 grown in TSS media, the carbon source used was either glucose or OPP, and 35 mL of these cultures were subjected to centrifugation at 10,000 × *g* for 10 min. The supernatant was passed through a 0.2 µm syringe filter (VWR, Radnor, PA, USA) and 1 mL of this sample (SUPERNATANT) was applied to each seed. For the cell pellet suspension, the pellet was suspended in TSS and then subjected to centrifugation again to remove spent media, and then resuspended in 35 mL of TSS. An aliquot (1 mL containing ~10^7^ CFU) of this sample (TSS-CELLS) was applied to each seed. The TSS broth culture (35 mL) without separating cells and supernatant was also prepared (TSS-CULTURE), and 1 mL of the broth culture was applied to each seed.

#### Preparation of *M. incognita* inoculum

*M. incognita* eggs were isolated and extracted from corn roots as described previously and 2,000 eggs/mL populations were inoculated into a 2 cm depth of soil in each cone-tainer (Stuewe & Sons, Tangent, OR, USA) during seed planting and covered with field soil. The *M. incognita-*inoculated soybean and cotton seeds were kept at room temperature in the greenhouse for 24 hr before transferring to a greenhouse growth chamber at 25 to 35°C.

#### Soil preparation and seed inoculation

Field soil (Malbis fine sandy loam 59% sand, 31% silt, 10% clay, <1% OM) was collected from the E.V. Smith Research Center of Auburn University (near Shorter, AL) and was mixed with sand at a ratio (2:1). In the greenhouse experiments, the soil/sand mix was placed into each 150 cm^3^ cone-tainers. Two soybean (DD VSG 75140) and two cotton seeds (DPL1558 NRB2RF) were placed into 2 cm depth of each cone-tainer to ensure seed germination. The different treatment group inocula (i.e. cell pellet suspensions, culture broths, or supernatants) of *B. velezensis* AP203 were applied on the soybean and cotton seed surface. The seeds were then covered with the soil/sand, kept at room temperature for 24 hr, before transferring to a greenhouse growth chamber (25 to 35°C).

### Statistical analyses

All data collected from the in vitro bioassay and greenhouse tests were analyzed with SAS 9.4 software (SAS Institute, Cary, NC, USA) using the PROC GLIMMIX (Generalized Linear Mixed Models) procedure that performs estimation and statistical inference for GLIMMIX at the *p* ≤ 0.05 level of significance. For the in vitro experiments, the mortality percentages of J2 of *M. incognita* were analyzed with four treatments and eight replicates. In the greenhouse experiment, plant height, root length, root and shoot fresh weight, and *M. incognita* eggs/plant data were collected and analyzed. The greenhouse experiments were arranged in a randomized complete block design (RCBD) with eleven treatments and eight replicates. All the in vitro and greenhouse experiments were repeated twice, and the data were pooled.

## Results

### In vitro antagonistic effects of *B. velezensis* AP203 with orange peel amendment

**Figure 1:**
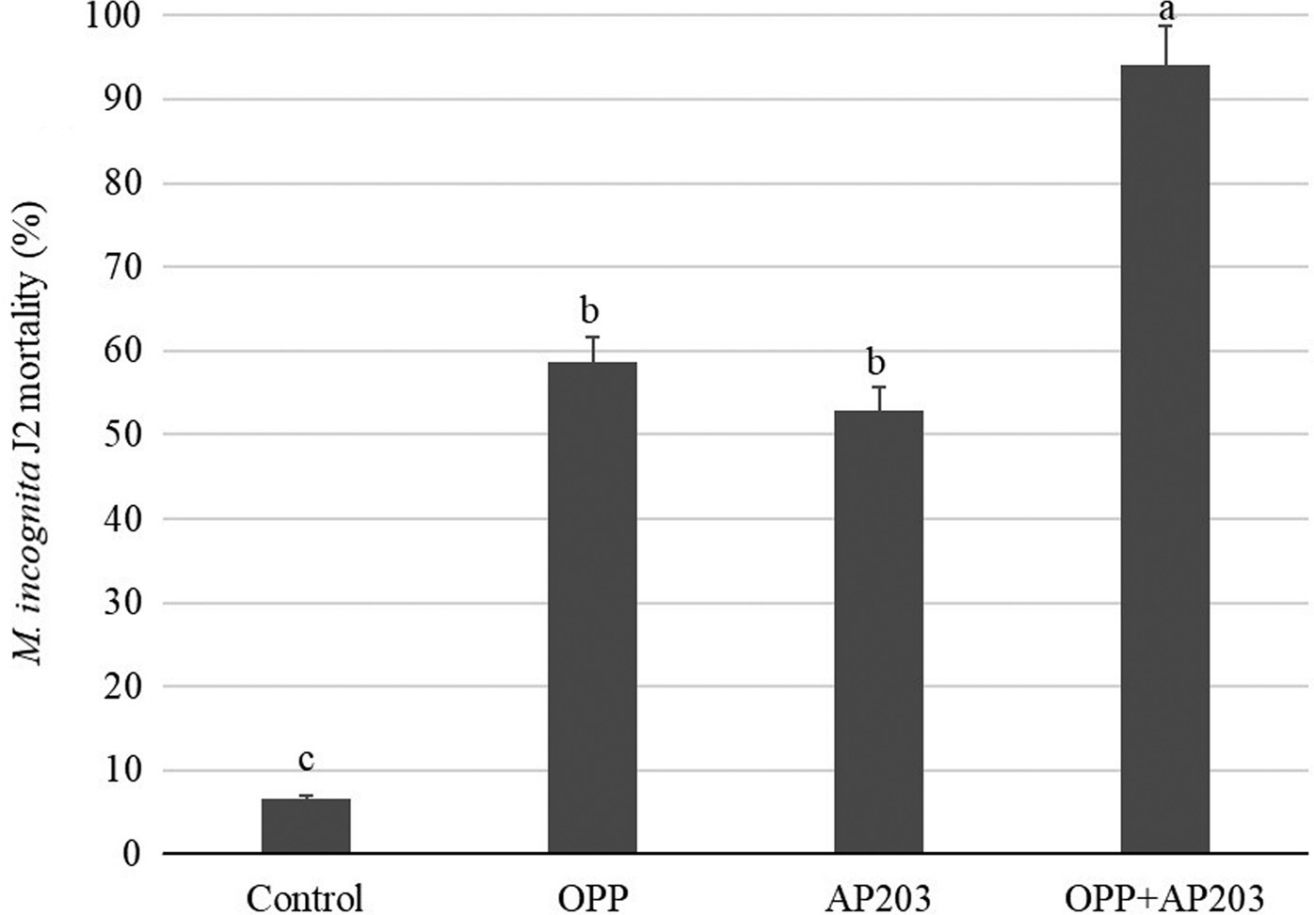
Effects of orange peel power (OPP) amendment on the mortality of J2 of *M. incognita* in vitro by *B. velezensis* AP203.

A spore preparation of *B. velezensis* AP203 with orange peel amendment was tested in vitro for its ability to kill *M. incognita* J2. The mortality percentage of *M. incognita* J2 ranged from 0 to 100%, with 94% mortality observed for *M. incognita* eggs inoculated with the combination of *B. velezensis* strain AP203 with 1.0% (w/v) OPP, which was significantly greater (*p* < 0.05) than that observed when eggs were incubated with *B. velezensis* AP203 alone (53%), 1.0% OPP (59%), or the negative control (7%) (Fig. 1). Interestingly, a significant increase in *M. incognita* J2 mortality relative to the negative control was also observed for *B. velezensis* AP203 alone (as had been observed previously) as well as inoculation with OPP alone.

### The effects of *B. velezensis* AP203 cultures grown in different media on *M. incognita* populations and cotton and soybean growth under greenhouse conditions

**Figure 2:**
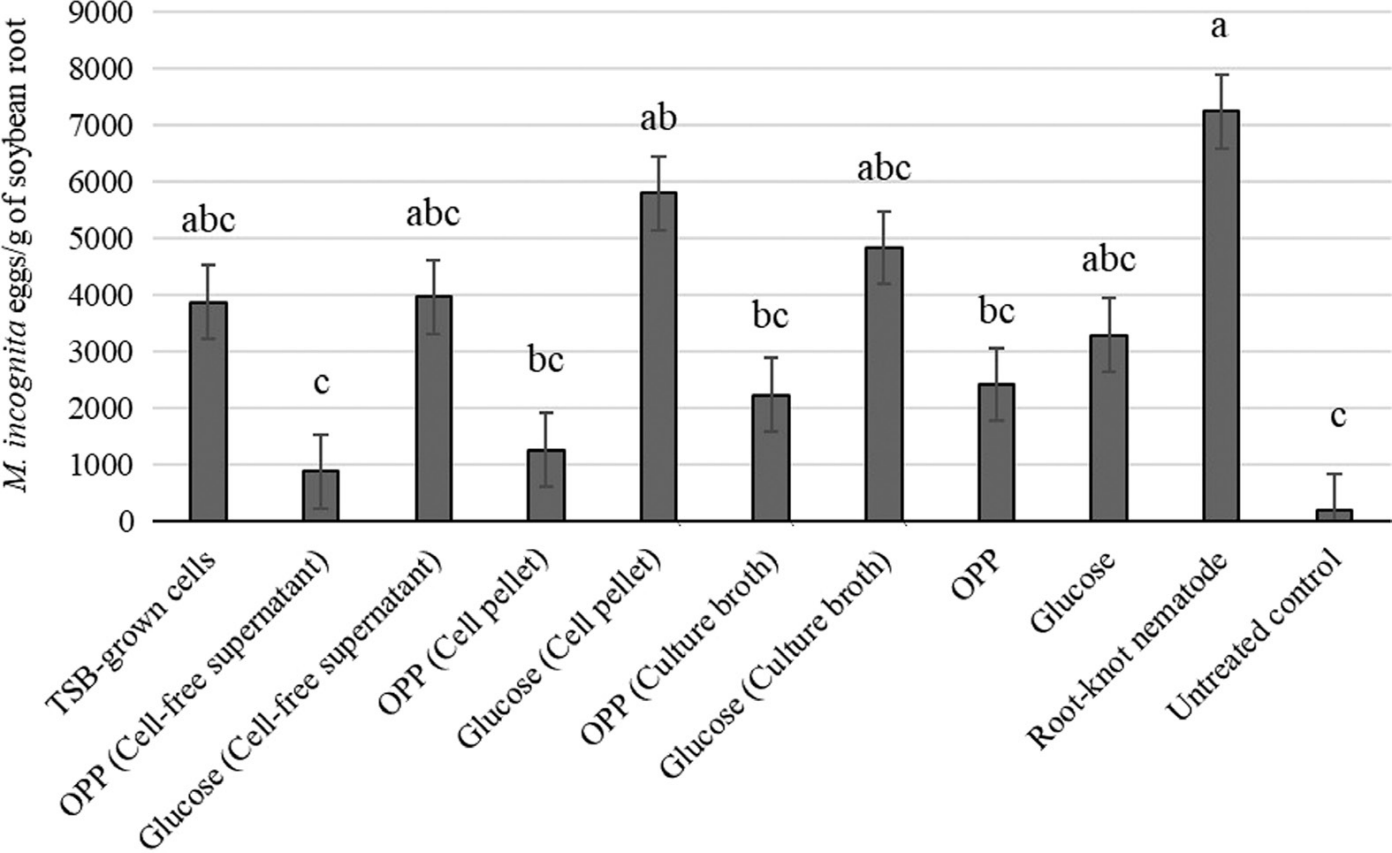
Effects of *B. velezensis* AP203 with an orange peel power (OPP) or glucose amendments on the number of *M. incognita* eggs on the roots of soybean at 45 days after planting in greenhouse trials.

**Figure 3:**
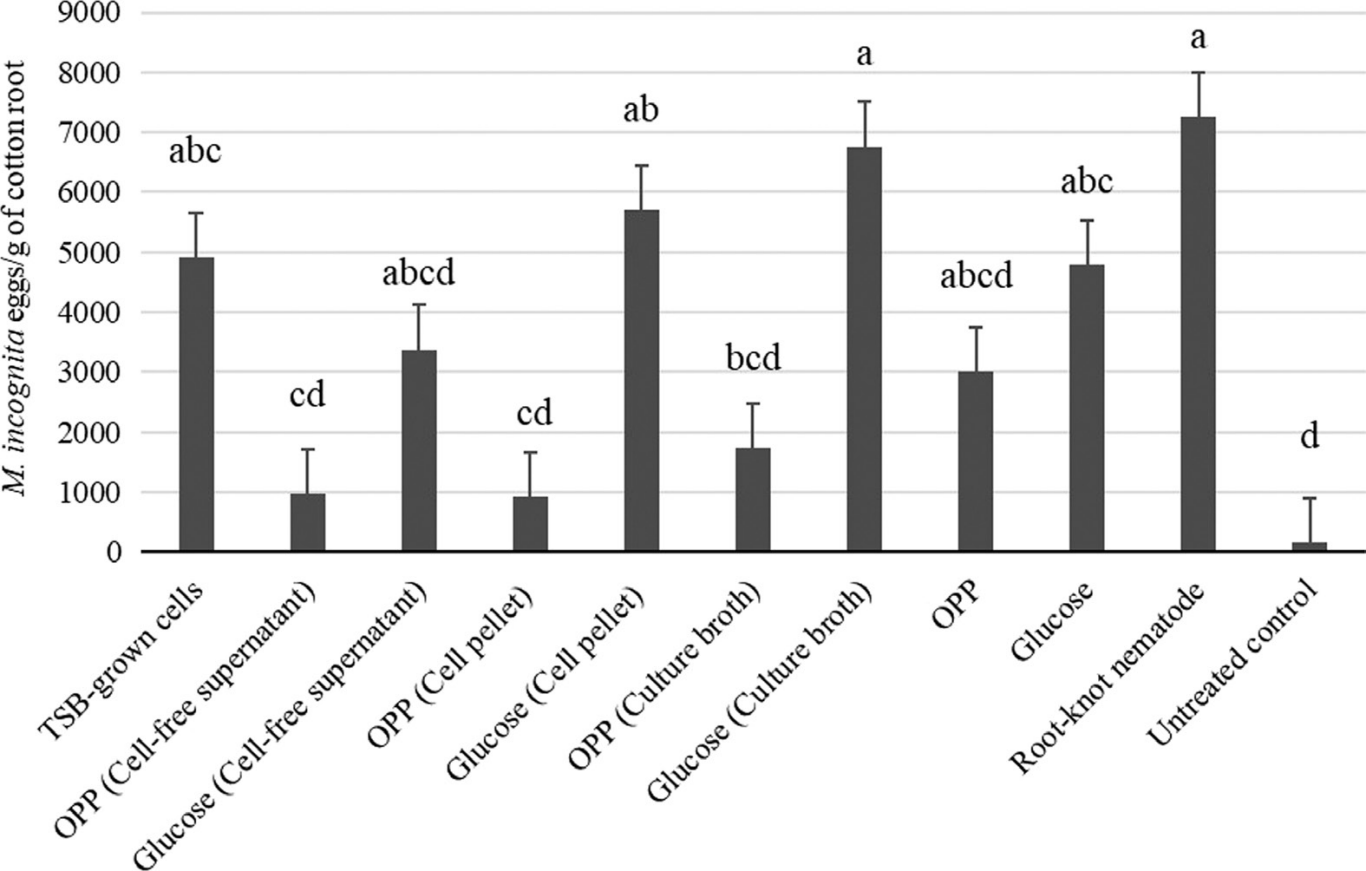
Effects of *B. velezensis* AP203 with an orange peel power (OPP) or glucose amendments on the number of *M. incognita* eggs on the roots of cotton at 45 days after planting in greenhouse trials.

Soybean root length of plants treated with culture broth from *B. velezensis* AP203 with OPP was significantly greater compared to the glucose treatments and *M. incognita* positive control treatment (Table 1). Soybean root length was also greater in the TSB-growth cells, OPP (supernatant) and OPP (culture) compared to the *M. incognita* control. OPP (culture) also significantly increased root fresh weight as compared to the *M. incognita* control (Table 1). Cotton shoot lengths of plants treated with cell pellets from *B. velezensis* AP203 with OPP was significantly greater compared to the glucose treatments and the *M. incognita* control (Table 2). Cotton root length was significantly increased by the OPP (supernatant), OPP (cells), and OPP (culture) compared to the *M. incognita* control (Table 2). Supernatant, cell pellet, or culture broth from *B. velezensis* AP203 with OPP had a maximum antagonistic activity against *M. incognita* eggs in soybean (Fig. 2) and cotton (Fig. 3) roots at 45 DAP. The *B. velezensis* AP203 with OPP (supernatant), OPP (cells), and OPP (culture) all reduced *M. incognita* populations as compared to the *M. incognita* grown on both crop plants without any additives. *B. velezensis* AP203 with glucose amended treatments (supernatant, cell pellet, culture broth from *B. velezensis AP203* with glucose) did not significantly reduce *M. incognita* populations compared to the *M. incognita* positive control treatment in soybean or cotton at 45 DAP (Figs. 2 and 3).

**Table 1.** Effect of *B. velezensis* AP203 amended with orange peel powder (OPP) or glucose on soybean growth at 45 days after planting in greenhouse trials.

Treatment^a^	Shoot length (cm)^b^		Root length (cm)		Shoot fresh weight (g)		Root fresh weight (g)	
TSB-grown cells	58.0	a	22.5	ab	6.51	ab	3.91	ab
OPP (supernatant)	67.4	a	21.9	abc	8.12	ab	4.20	ab
Glucose (supernatant)	66.8	a	19.9	cde	7.55	ab	3.98	ab
OPP (Cells)	72.3	a	22.0	abc	9.06	ab	4.92	a
Glucose (Cells)	62.5	a	20.1	bcde	8.43	ab	3.42	ab
OPP (Culture)	72.0	a	22.6	a	10.20	a	4.11	ab
Glucose (Culture)	60.8	a	19.0	e	8.25	ab	4.00	ab
OPP	62.5	a	21.4	abcd	8.12	ab	3.65	ab
Glucose	67.9	a	20.0	cde	5.95	ab	2.53	b
*M. incognita* nematode								
Untreated control								

**Notes:** ^a^ *B. velezensis* AP203 grown in 1.0% (w/v) OPP or glucose-amended TSS medium. *M. incognita* 2,000 eggs/150 cm^3^ soil was added to all plants except the untreated control; ^b^means with the same letter are not significantly different at *p* ≤ 0.05 level of significance.

**Table 2.** Effect of *B. velezensis* AP203, amended orange peel powder (OPP) or glucose on cotton growth at 45 days after planting in greenhouse trials.

	Shoot length		Root length		Shoot fresh		Root fresh	
Treatment^a^	(cm)^b^		(cm)		weight (g)		weight (g)	
TSB-grown cells	26.0	ab	18.5	abc	2.6	a	1.7	a
OPP (supernatant)	27.3	ab	20.2	ab	2.5	a	1.7	a
Glucose (supernatant)	26.8	ab	18.2	abc	2.5	a	1.6	a
OPP (Cells)	30.7	a	21.2	ab	2.2	a	1.9	a
Glucose (Cells)	27.0	ab	16.1	bc	2.7	a	1.6	a
OPP (Culture)	30.6	ab	20.1	ab	2.4	a	1.4	a
Glucose (Culture)	28.8	ab	18.1	abc	2.5	a	1.6	a
OPP	23.2	ab	17.7	abc	2.1	a	1.7	a
Glucose	22.6	b	16.1	bc	2.3	a	1.1	a
*M. incognita* nematode	20.0	b	14.3	c	1.7	a	0.9	a
Untreated control	26.2	ab	21.8	a	2.7	a	1.3	a

**Notes:** ^a^ *B. velezensis* AP203 grew in 1.0% (w/v) OPP or glucose-amended TSS medium. *M. incognita* 2,000 eggs/150 cm^3^ soil was added to all plants except the untreated control; ^b^means with the same letter are not significantly different at *p* ≤ 0.05 level of significance.

### Secretion of secondary metabolites by *B. velezensis* AP203 is affected by growth on orange peel powder

**Figure S1:**
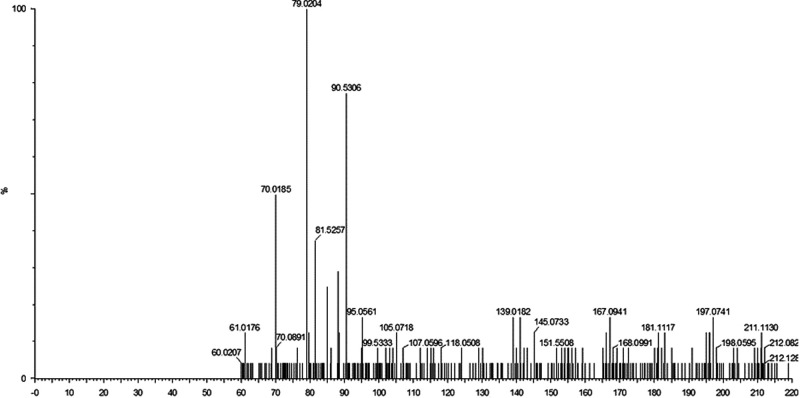
LC-MS/MS spectra of the peaks eluted at 6.69 min (m/z of 211.11).

**Figure S2:**
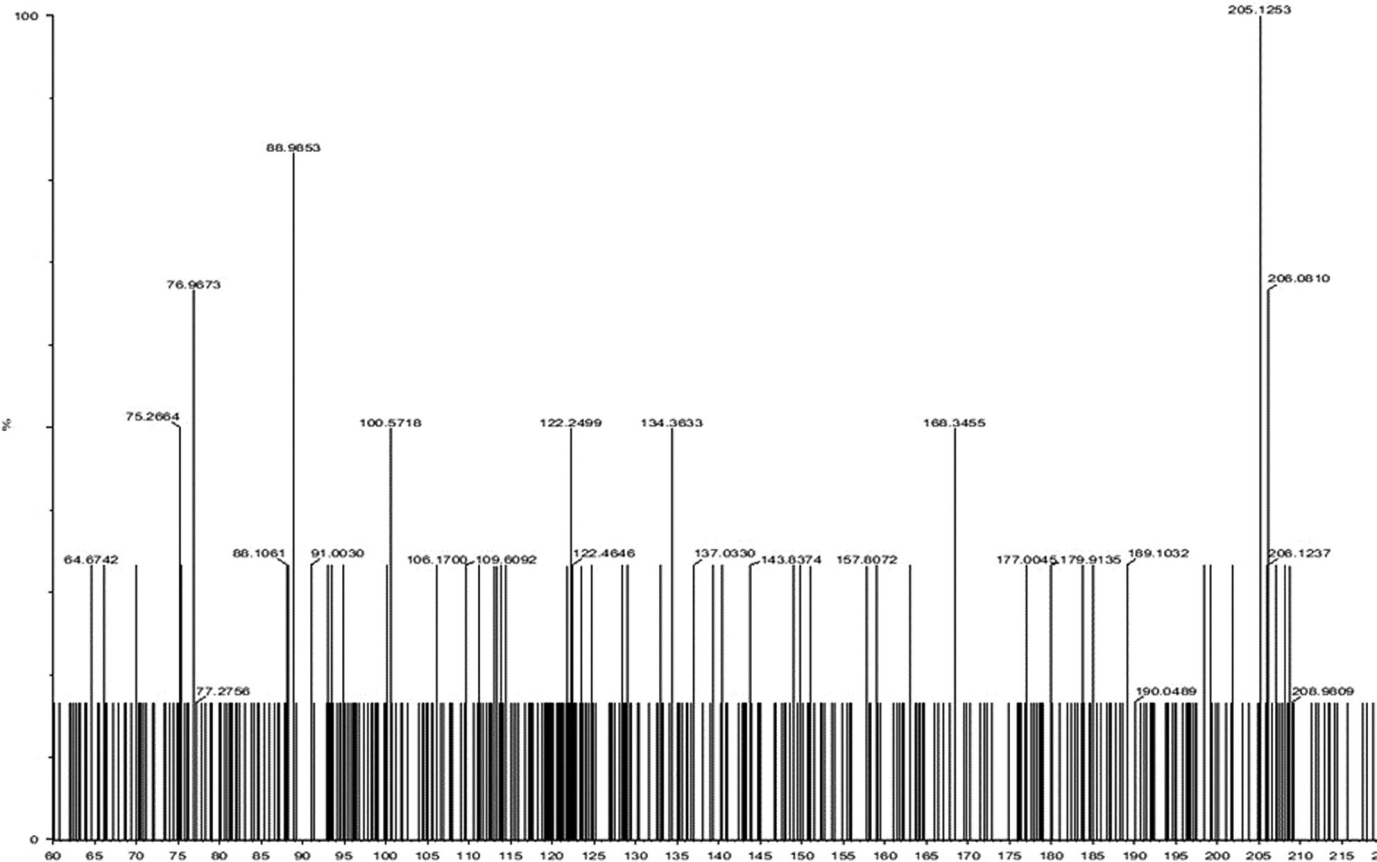
LC-MS/MS spectra of the peaks eluted at 2.40 min (m/z of 206.12).

**Figure S3:**
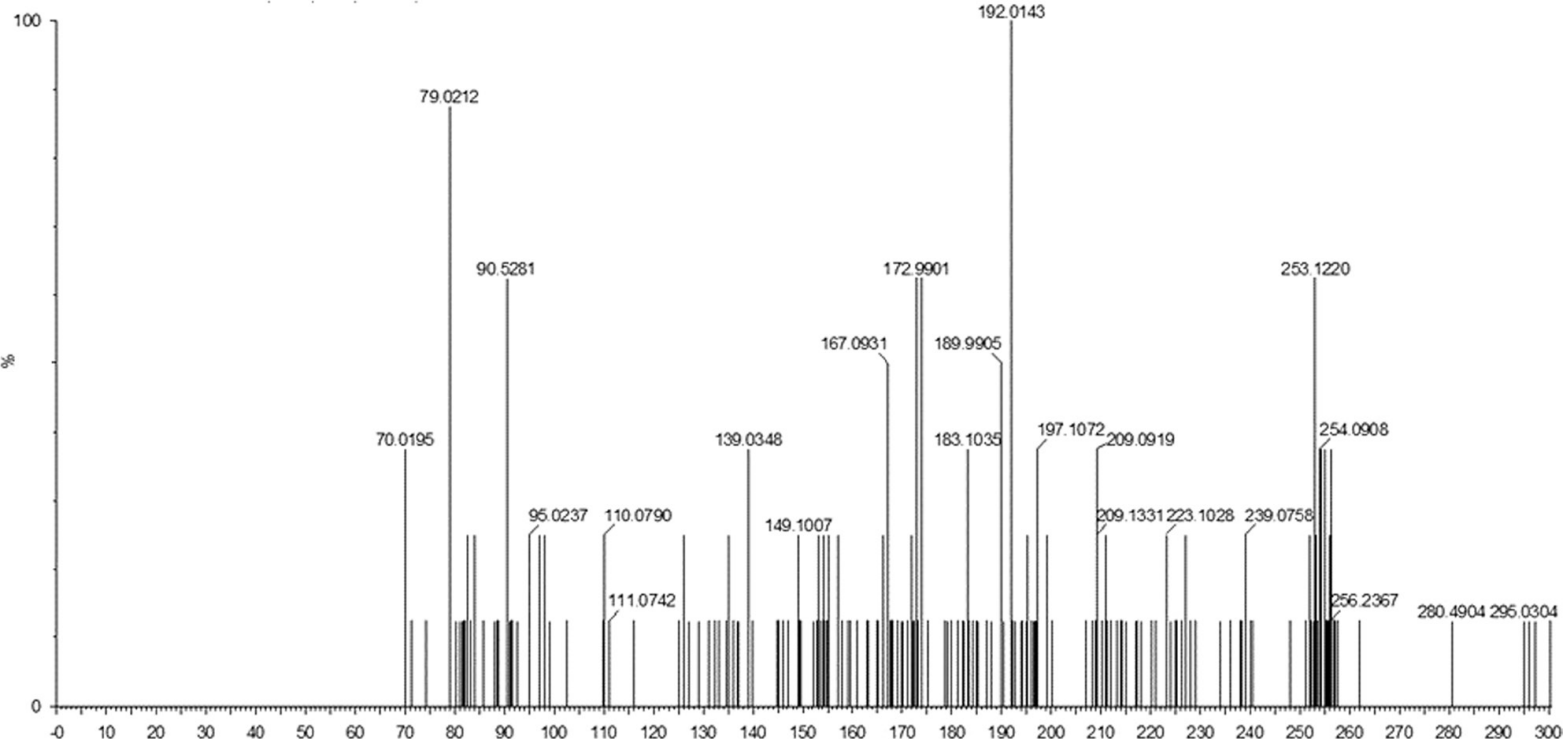
LC-MS/MS spectra of the peaks eluted at 6.63 min (m/z of 253.12) and 3.39 min (m/z of 295.17).

Numerous secondary metabolites were detected in the supernatant of *B. velezensis* AP203 amended with orange peel, which revealed a complex mass spec profile even after removing the metabolites present in orange peel powder (data not shown). Among the mass ions predicted to be expressed by *B. velezensis* AP203, and not present in orange peel, were four metabolites that had previously been reported to have nematicidal activity: (i) 1,3-Diphenyl-2-propanone, (ii) p-(3,4-Dihydro-6-methoxy-2-naphthyl) phenol, (iii) (E)-1,1,-(1,2-Diethyl-1,2-ethenediyl) bis (4-methoxybenzene), and (iv) 3-(Dimethylamino) propyl benzoate (Table 3) (Huang et al., 2010). The retention times (RT) of these secondary metabolites were 6.69, 6.63, 3.39, and 2.40 min, respectively (Table 3). The product mass to ions charge ratio (m/z) of these secondary metabolites were 211.11, 253.12, 295.17, and 206.12, respectively (Table 3 and Figs. S1-S3). The relative abundances (RA) per colony forming units (CFU) of secondary metabolite 1,3-Diphenyl-2-propanone were 21.11, 25.31, 29.51, and 20.61 (Table 3). The relative abundance (RA) per CFU indicated that (E)-1,1,-(1,2-Diethyl-1,2-ethenediyl) bis (4-methoxybenzene) was produced most abundantly under these culture conditions when grown with OP as a carbon source, followed by p-(3,4-Dihydro-6-methoxy-2-naphthyl) phenol, 1,3-Diphenyl-2-propanone, and 3-(Dimethylamino) propyl benzoate in decreasing order of RA/CFU (Table 3). The complete list of metabolites detected from the supernatant of *B. velezensis* AP203 grown on orange peel in TSS minimal medium are listed in Supplementary Table 4.

**Table 3.** Predicted secondary metabolites found in cell-free supernatants of *B. velezensis* AP203 after 48 hr growth in 0.5% (w/v) OPP amended Tris Spizizen Salts (TSS) media.

Treatment^a^	RT (min)^b^	Product ions (m/z)	RA/CFU^c^	Predicted metabolites
OPAP203 1	6.69 min	211.11	21.11	1,3-Diphenyl-2-propanone
OPAP203 2	6.63 min	253.12	25.31	p-(3,4-Dihydro-6-methoxy-2-naphthyl) phenol
OPAP203 3	3.39 min	295.17	29.51	(E)-1,1,-(1,2-Diethyl-1,2-ethenediyl) bis (4-methoxybenzene)
OPAP203 4	2.40 min	206.12	20.61	3-(Dimethylamino) propyl benzoate
GluAP203	0	0	0	0
OPP	0	0	0	0
Glucose	0	0	0	0

**Notes:** ^a^The in vitro *B.velezensis* AP203 growth test was repeated twice; ^b^RT indicates retention time; ^c^RA indicates relative abundance/colony forming units.

**Table S1:** List of predicted secondary metabolites found in cell-free supernatants of *B. velezensis* AP203 after 48 hr growth in 0.5% (w/v) orange peel power (OPP) amended Tris Spizizen Salts (TSS) media.

Query ID	Query m/z	Name of the bioactive compounds	Formula	Exact mass
1	101.0711	Gyromitrin;Acetaldehyde methylformylhydrazone	C4H8N2O	100.0636629
2	103.0559	Indoleacetic acid	C10H9NO2	175.0633285
3	103.0559	5-Hydroxyindoleacetaldehyde	C10H9NO2	175.0633285
4	104.0549	2-Ethyl-1-hexanol, 9CI; (Â±)-form, O-Sulfate	C8H18O4S	210.0925798
5	104.0568	2-Ethyl-1-hexanol, 9CI; (Â±)-form, O-Sulfate	C8H18O4S	210.0925798
7	104.0585	Histidinyl-Glycine	C8H12N4O3	212.0909403
8	104.0585	D-Glycero-D-galacto-heptitol	C7H16O7	212.0896029
9	104.0585	Glycyl-Histidine	C8H12N4O3	212.0909403
10	104.0707	N-methyl-beta-alanine	C4H9NO2	103.0633285
11	104.0707	(2S)-2-Nitrobutane	C4H9NO2	103.0633285
13	104.0707	Ethyl carbamic acid methyl ester	C4H9NO2	103.0633285
14	104.0707	N-Methyl-L-alanine	C4H9NO2	103.0633285
15	104.0707	HBA	C4H9NO2	103.0633285
16	104.0707	DL-3-aminobutyrate	C4H9NO2	103.0633285
17	104.0707	Mefenamic acid	C15H15NO2	241.1102787
18	104.0707	N-[2-(4-Hydroxyphenyl)ethyl]benzamide	C15H15NO2	241.1102787
19	104.0707	2-Amino-2-methylpropanoate;2-Aminoisobutyric acid	C4H9NO2	103.0633285
20	104.0707	(R,S)-3-Amino-2-methylpropanoate	C4H9NO2	103.0633285
21	104.0707	beta-alanine-methyl-ester	C4H9NO2	103.0633285
22	104.0707	N,N-Dimethylglycine;Dimethylglycine	C4H9NO2	103.0633285
23	104.0707	N-Ethylglycine	C4H9NO2	103.0633285
24	104.0707	(R)-2-Aminobutanoic acid;(S)-2-Aminobutanoate	C4H9NO2	103.0633285
25	104.0707	1-nitrobutane	C4H9NO2	103.0633285
26	104.0707	4-Aminobutanoate;4-Aminobutanoic acid	C4H9NO2	103.0633285
27	105.0366	3-methylthiopropanal	C4H8OS	104.0295856
28	105.0366	Acutifolane A	C16H22O3	262.1569
29	105.0366	tetrahydrothiophene 1-oxide	C4H8OS	104.0295856
30	105.0367	3-methylthiopropanal	C4H8OS	104.0295856
31	105.0367	tetrahydrothiophene 1-oxide	C4H8OS	104.0295856
32	105.0372	3-methylthiopropanal	C4H8OS	104.0295856
33	105.0372	tetrahydrothiophene 1-oxide	C4H8OS	104.0295856
34	105.0376	3-methylthiopropanal	C4H8OS	104.0295856
35	105.0376	tetrahydrothiophene 1-oxide	C4H8OS	104.0295856
37	105.044	3-cyanopyridine	C6H4N2	104.0374481
38	105.044	2-Cyanopyridine	C6H4N2	104.0374481
39	105.044	4-Cyanopyridine	C6H4N2	104.0374481
40	105.0441	(+)-18-Hydroxy-7,16-sacculatadiene-11,12-dial	C20H30O3	318.2195
41	105.0441	ent-7alpha-hydroxykaur-16-en-19-oic acid	C20H30O3	318.2195
42	105.0441	2-Cyanopyridine	C6H4N2	104.0374481
43	105.0441	Oxymesterone	C20H30O3	318.2194948
44	105.0441	4-Cyanopyridine	C6H4N2	104.0374481
45	105.0441	3-cyanopyridine	C6H4N2	104.0374481
46	105.0441	8-oxo-5E,9Z,11Z,14Z-eicosatetraenoic acid	C20H30O3	318.2195
47	105.0441	9-oxo-5E,7Z,11Z,14Z-eicosatetraenoic acid	C20H30O3	318.2195
48	105.0441	11-oxo-5E,8Z,12Z,14Z-Eicosatetraenoic acid	C20H30O3	318.2195
49	105.0441	(+)-7beta-Hydroxy-15-beyeren-19-oic acid	C20H30O3	318.2195
50	105.0557	Tyrosyl-Tyrosine	C18H20N2O5	344.1372218
51	105.0651	Aminoserine	C3H8N2O2	104.0585775
52	105.0651	L-2,3-Diaminopropionate	C3H8N2O2	104.0585775
53	105.0651	Hydroxyaminoalanine	C3H8N2O2	104.0585775
54	105.0662	2,3-Diaminopropanoic acid	C3H8N2O2	104.0585775
55	105.0672	4,-O-Methylbavachalcone	C22H24O4	352.1675
56	105.0672	Ovalichalcone	C22H24O4	352.1675
57	105.0672	Pongagallone A	C22H24O4	352.1675
58	105.0672	Candidone	C22H24O4	352.1675
59	105.0672	Methylhildgardtol A	C22H24O4	352.1675
60	105.0672	Methylhildgardtol B	C22H24O4	352.1675
61	105.0672	Xuulanin	C22H24O4	352.1675
62	105.0715	Valganciclovir	C14H22N6O5	354.1651678
63	105.0733	12-hydroxyjasmonic acid 12-O-beta-D-glucoside	C19H30O8	386.1941
64	105.0733	Citroside A	C19H30O8	386.1940679
65	105.0733	6,9-Dihydroxy-4,7-megastigmadien-3-one	C19H30O8	386.1940679
66	107.0845	p-Xylene;1,4-Dimethylbenzene;p-Methyltoluene	C8H10	106.0782503
67	107.0845	Ethylbenzene;Phenylethane;Ethylbenzol;Ethylenzene	C8H10	106.0782503
68	107.0845	o-Xylene;o-Dimethylbenzene;o-Methyltoluene	C8H10	106.0782503
69	107.0845	m-Xylene;1,3-Dimethylbenzene;1,3-Xylene	C8H10	106.0782503
70	107.0848	o-Xylene;o-Dimethylbenzene;o-Methyltoluene	C8H10	106.0782503
71	107.0848	m-Xylene;1,3-Dimethylbenzene;1,3-Xylene	C8H10	106.0782503
72	107.0848	p-Xylene;1,4-Dimethylbenzene;p-Methyltoluene	C8H10	106.0782503
73	107.0848	Ethylbenzene;Phenylethane;Ethylbenzol;Ethylenzene	C8H10	106.0782503
74	107.0848	O-6-deoxy-a-L-galactopyranosyl	C20H33NO14	511.1901048
75	107.0851	o-Xylene;o-Dimethylbenzene;o-Methyltoluene	C8H10	106.0782503
76	107.0851	m-Xylene;1,3-Dimethylbenzene;1,3-Xylene	C8H10	106.0782503
77	107.0851	p-Xylene;1,4-Dimethylbenzene;p-Methyltoluene	C8H10	106.0782503
78	107.0851	Ethylbenzene;Phenylethane;Ethylbenzol;Ethylenzene	C8H10	106.0782503
79	107.0858	p-Xylene;1,4-Dimethylbenzene;p-Methyltoluene	C8H10	106.0782503
80	107.0858	Ethylbenzene;Phenylethane;Ethylbenzol;Ethylenzene	C8H10	106.0782503
81	107.0858	o-Xylene;o-Dimethylbenzene;o-Methyltoluene	C8H10	106.0782503
82	107.0858	m-Xylene;1,3-Dimethylbenzene;1,3-Xylene	C8H10	106.0782503
83	107.0858	Daunorubicin	C27H29NO10	527.1791462
84	107.0859	m-Xylene;1,3-Dimethylbenzene;1,3-Xylene	C8H10	106.0782503
85	107.0859	p-Xylene;1,4-Dimethylbenzene;p-Methyltoluene	C8H10	106.0782503
86	107.0859	Ethylbenzene;Phenylethane;Ethylbenzol;Ethylenzene	C8H10	106.0782503
87	107.0859	o-Xylene;o-Dimethylbenzene;o-Methyltoluene	C8H10	106.0782503
88	109.0287	1,2-Benzoquinone	C6H4O2	108.0211294
89	109.0287	Quinone;p-Benzoquinone;Chinone	C6H4O2	108.0211294
90	109.0306	Hordatine B glucoside	C35H50N8O10	742.3649899
91	109.0306	Hydroxymethylmethylsilanediol	C2H8O3Si	108.0242707
92	109.0309	Hydroxymethylmethylsilanediol	C2H8O3Si	108.0242707
93	109.0309	Monothioglycerol	C3H8O2S	108.0245002
94	109.0315	Hydroxymethylmethylsilanediol	C2H8O3Si	108.0242707
95	109.0316	Hydroxymethylmethylsilanediol	C2H8O3Si	108.0242707
96	109.0316	Hydroxymethylmethylsilanediol	C2H8O3Si	108.0242707
97	109.0321	Hydroxymethylmethylsilanediol	C2H8O3Si	108.0242707
98	109.0642	Benzenemethanol;Phenylmethanol;Phenylcarbinol	C7H8O	108.0575149
99	109.0642	o-Cresol;2-Hydroxytoluene;o-Methylphenol	C7H8O	108.0575149
100	109.0642	3-Cresol;m-Cresol;3-Hydroxytoluene	C7H8O	108.0575149
101	109.0642	4-Cresol;p-Cresol;4-Hydroxytoluene	C7H8O	108.0575149
102	109.0642	Anisole;Methoxybenzene;Methyl phenyl ether	C7H8O	108.0575149
103	111.0434	Resorcinol;Resorcin;1,3-Benzenediol	C6H6O2	110.0367794
104	111.0434	Hydroquinone;p-Benzenediol;1,4-Benzenediol	C6H6O2	110.0367794
105	111.0434	5-Methyl-2-furaldehyde;5-Methyl-2-furfural	C6H6O2	110.0367794
106	111.0434	o-Benzosemiquinone	C6H6O2	110.0367794
107	111.0434	Catechol;1,2-Benzenediol;o-Benzenediol	C6H6O2	110.0367794
108	111.0434	Benzosemiquinone;p-Benzosemiquinone	C6H6O2	110.0367794
109	111.0437	Resorcinol;Resorcin;1,3-Benzenediol	C6H6O2	110.0367794
110	111.0437	Hydroquinone;p-Benzenediol;1,4-Benzenediol	C6H6O2	110.0367794
111	111.0437	5-Methyl-2-furaldehyde;5-Methyl-2-furfural	C6H6O2	110.0367794
112	111.0437	Catechol;1,2-Benzenediol;o-Benzenedio	C6H6O2	110.0367794
113	111.0437	Benzosemiquinone;p-Benzosemiquinone	C6H6O2	110.0367794
114	111.0438	2-Furanmethanol	C5H6O2	98.03677944
115	111.0438	Benzosemiquinone;p-Benzosemiquinone	C6H6O2	110.0367794
116	111.0438	Resorcinol;Resorcin;1,3-Benzenediol	C6H6O2	110.0367794
117	111.0438	Hydroquinone;p-Benzenediol;1,4-Benzenediol	C6H6O2	110.0367794
118	111.0438	5-Methyl-2-furaldehyde;5-Methyl-2-furfural	C6H6O2	110.0367794
119	111.0438	o-Benzosemiquinone	C6H6O2	110.0367794
120	111.0438	penta-2,4-dienoic acid;beta-vinyl acrylic acid	C5H6O2	98.0368
121	111.0438	Catechol;1,2-Benzenediol;o-Benzenediol	C6H6O2	110.0367794
122	111.0438	5-Methyl-2(3H)-furanone	C5H6O2	98.03677944
123	111.0447	o-Benzosemiquinone	C6H6O2	110.0367794
124	111.0447	Catechol;1,2-Benzenediol;o-Benzenediol	C6H6O2	110.0367794
125	111.0447	Benzosemiquinone;p-Benzosemiquinone	C6H6O2	110.0367794
126	111.0447	Resorcinol;Resorcin;1,3-Benzenediol;1,3-Dihydroxybenzene	C6H6O2	110.0367794
127	111.0447	Hydroquinone;p-Benzenediol;1,4-Benzenediol	C6H6O2	110.0367794
128	111.0447	5-Methyl-2-furaldehyde;5-Methyl-2-furfural	C6H6O2	110.0367794
129	111.0448	o-Benzosemiquinone	C6H6O2	110.0367794
130	111.0448	Catechol;1,2-Benzenediol;o-Benzenediol	C6H6O2	110.0367794
131	111.0448	Benzosemiquinone;p-Benzosemiquinone	C6H6O2	110.0367794
132	111.0448	Resorcinol;Resorcin;1,3-Benzenediol;1,3-Dihydroxybenzene	C6H6O2	110.0367794
133	111.0448	Hydroquinone;p-Benzenediol;1,4-Benzenediol	C6H6O2	110.0367794
134	111.0451	Catechol;1,2-Benzenediol;o-Benzenediol	C6H6O2	110.0367794
135	111.0451	Benzosemiquinone;p-Benzosemiquinone	C6H6O2	110.0367794
136	111.0451	Resorcinol;Resorcin;1,3-Benzenediol;1,3-Dihydroxybenzene	C6H6O2	110.0367794
137	111.0451	Hydroquinone;p-Benzenediol;1,4-Benzenediol	C6H6O2	110.0367794
138	111.0451	5-Methyl-2-furaldehyde;5-Methyl-2-furfural	C6H6O2	110.0367794
139	121.0316	Dimethylsulfonioacetate	C4H8O2S	120.0245002
140	121.0316	3-(Methylthio)propionic acid;3-Methylthiopropionate	C4H8O2S	120.0245002
141	121.0316	sulfolane	C4H8O2S	120.0245002
142	121.032	Dimethylsulfonioacetate	C4H8O2S	120.0245002
143	121.032	3-(Methylthio)propionic acid;3-Methylthiopropionate	C4H8O2S	120.0245002
144	121.032	sulfolane	C4H8O2S	120.0245002
145	121.0324	Dimethylsulfonioacetate	C4H8O2S	120.0245002
146	121.0324	3-(Methylthio)propionic acid;3-Methylthiopropionate	C4H8O2S	120.0245002
147	121.0324	sulfolane	C4H8O2S	120.0245002
148	121.0325	Dimethylsulfonioacetate	C4H8O2S	120.0245002
149	121.0325	3-(Methylthio)propionic acid;3-Methylthiopropionate	C4H8O2S	120.0245002
150	121.0325	sulfolane	C4H8O2S	120.0245002
151	121.0325	Dimethylsulfonioacetate	C4H8O2S	120.0245002
152	121.0325	3-(Methylthio)propionic acid;3-Methylthiopropionate	C4H8O2S	120.0245002
153	121.0325	sulfolane	C4H8O2S	120.0245002
154	121.0325	Dimethylsulfonioacetate	C4H8O2S	120.0245002
155	121.0325	3-(Methylthio)propionic acid;3-Methylthiopropionate	C4H8O2S	120.0245002
156	121.0325	sulfolane	C4H8O2S	120.0245002
157	121.037	3-nitro-1-propionate	C3H6NO4	120.0296827
158	121.0379	3-nitro-1-propionate	C3H6NO4	120.0296827
159	121.05	2,3-dihydroxy-2-methyl-propanoic acid	C4H8O4	120.0422587
160	121.05	L-(+)-Erythrose;D-threo-Aldose;D-Erythrulose	C4H8O4	120.0422587
161	121.05	3-Deoxytetronic acid	C4H8O4	120.0422587
162	121.05	4-Deoxyerythronic acid	C4H8O4	120.0422587
163	121.05	L-Erythrulose;L-glycero-Tetrulose	C4H8O4	120.0422587
164	121.05	3,4-Dihydroxybutyric acid	C4H8O4	120.0422587
165	121.05	D-Threose;D-threo-Tetrose;D-Erythrose	C4H8O4	120.0422587
166	121.0516	Purine	C5H4N4	120.0435961
167	122.0962	3,4-DIMETHYLANILINE	C8H11N	121.0891494
168	122.0962	1-Phenylethylamine;alpha-Phenylethylamine	C8H11N	121.0891494
169	122.0962	N-Ethylaniline;N-Ethylbenzenamine	C8H11N	121.0891494
170	122.0962	Phenethylamine;2-Phenylethylamine;beta-Phenylethylamine	C8H11N	121.0891494
171	122.0962	2,4-Dimethylaniline;2,4-DMA	C8H11N	121.0891494
172	122.0962	N,N-Dimethylaniline; N,N-Dimethylbenzenamine	C8H11N	121.0891494
173	122.0965	1-Phenylethylamine;alpha-Phenylethylamine	C8H11N	121.0891494
174	122.0965	2,6-Dimethylaniline	C8H11N	121.0891494
175	122.0965	N-Ethylaniline;N-Ethylbenzenamine	C8H11N	121.0891494
176	122.0965	2,5-Dimethylalanine	C8H11N	121.0891494
177	122.0965	2-Phenylethylamine;beta-Phenylethylamine	C8H11N	121.0891494
178	181.0694	Sorbose;xylo-Hexulose;D-Fructose	C6H12O6	180.0633881
179	181.0694	2-Deoxy-D-gluconate	C6H12O6	180.0633881
180	181.0694	Ketose	C6H12O6	180.0633881
181	181.0996	Methylphophonic acid diisopropyl ester	C7H17O3P	180.0915309
182	229.1235	1,1-Bis(4-hydroxyphenyl)propane	C15H16O2	228.1150298
183	229.1235	Mansonone C	C15H16O2	228.1150298
184	229.1235	Bisphenol A;2,2-Bis(4-Hydroxyphenyl)propane	C15H16O2	228.1150298
185	229.1235	dihydropinosylvin monomethylether	C15H16O2	228.1150298
186	229.1285	Deoxyguanidinoproclavaminic acid	C9H16N4O3	228.1222404
187	229.1298	Deoxyamidinoproclavaminate	C9H16N4O3	228.1222404
188	230.058	Lamivudine;3TC;2’,3’-Dideoxy-3’-thiacytidine	C8H11N3O3S	229.0521119
189	230.058	Carbonylphophonic acid	C9H12NO4P	229.0503944
190	230.058	2,3-Dihydroxy-2’-carboxybiphenyl	C13H9O4-	229.0500838
191	357.1718	Rutamarin	C21H24O5	356.1623739
191	357.1718	Gingerenone A	C21H24O5	356.1623739
192	357.1718	alpha,beta-dihydroxanthohumol	C21H24O5	356.1623739
193	357.1718	Kadsurenone;Denudatin B	C21H24O5	356.1623739
194	371.0795	Rebamipide	C19H15ClN2O4	370.0720347
195	371.0795	Digalacturonate;Digalacturonic acid	C12H18O13	370.0747407
196	371.0795	1,2-beta-D-Glucuronosyl-D-glucuronate	C12H18O13	370.0747407
197	371.145	Napththalene-2-sulfonamide	C20H22N2O3S	370.1351133
198	371.1501	iso-dehydrocycloxanthohumol hydrate	C21H22O6	370.1416384
199	371.1501	xanthohumol D	C21H22O6	370.1416384
200	371.1501	5’-Prenylhomoeriodictyol;Sigmoidin B 3’-methyl ether	C21H22O6	370.1416384
201	371.1501	xanthohumol B	C21H22O6	370.1416384
202	371.1501	curcumin	C21H22O6	370.1416384
203	371.1501	Alkannin beta,beta-dimethylacrylate	C21H22O6	370.1416384
204	371.1501	Sophoraisoflavanone A	C21H22O6	370.1416384
205	371.1509	iso-dehydrocycloxanthohumol hydrate	C21H22O6	370.1416384
206	371.1509	xanthohumol D	C21H22O6	370.1416384
207	371.1509	5’-Prenylhomoeriodictyol;Sigmoidin B 3’-methyl ether	C21H22O6	370.1416384
208	371.1509	xanthohumol B	C21H22O6	370.1416384
209	371.1509	curcumin	C21H22O6	370.1416384
210	371.1509	Alkannin beta,beta-dimethylacrylate	C21H22O6	370.1416384
211	371.1509	Sophoraisoflavanone A	C21H22O6	370.1416384
212	371.1528	Galactan;Amylose	C14H26O11	370.1475117
213	371.1528	Galactan;Amylose	C14H26O11	370.1475117
214	371.1541	Galactan;Amylose	C14H26O11	370.1475117
215	404.1365	trifluoperazine	C21H20F3N3S	403.1330029
216	404.1484	Ampicillin trihydrate	C16H25N3O7S	403.1413209
217	405.1191	Spectinomycin dihydrochloride	C14H26Cl2N2O7	404.1117066
218	405.1267	Sulfinpyrazone;Sulfoxyphenylpyrazolidine	C23H20N2O3S	404.1194632
219	405.1269	Sulfinpyrazone;Sulfoxyphenylpyrazolidine	C23H20N2O3S	404.1194632

## Discussion

This study demonstrated that the PGPR *B. velezensis* AP203 when used in combination with a pectin-rich orange peel amendment resulted in significantly enhanced *M. incognita* J2 mortality. The nematicidal activity observed under laboratory and greenhouse conditions suggests that there are nematicidal secondary metabolites produced by *B. velezensis* AP203 in the presence of a pectin-rich orange peel growth substrate. In the presence of different carbohydrate substrates, the expression of secondary metabolites by PGPR strains has been previously shown to vary considerably (Zhu et al., 2013). The production of surfactin by *B. velezensis* SQY162 (previously known as *B. amyloliquefaciens* subsp. *plantarum*) was significantly increased when this strain was inoculated with an exogenous pectin amendment, resulting in enhanced biological control efficacy against bacterial wilt of tobacco caused by *Ralstonia solanacearum* (Wu et al., 2015). Previous studies have documented that *B. velezensis* strains have pectinolytic activity and can utilize pectin as a sole carbon and energy source (Hossain et al., 2015). Hence, exogenous pectin amendments serve as a prebiotic growth substrate that can be utilized by *B. velezensis* or other pectinolytic microorganisms, thereby enhancing the metabolic activities of the bacteria, which in the case of PGPR *B. velezensis* strains can include plant growth-promotion and biofilm formation (Beauregard et al., 2013; Hassan et al., 2019). In a similar study, it was reported that chitinolytic bacteria provided with a chitin substrate significantly reduced soybean cyst nematode (*Heterodera glycines*) populations under greenhouse conditions (Tian et al., 2000). Collectively, these previous studies and the results of this study support the hypothesis that the combination of a prebiotic complex carbohydrate (e.g. pectin-rich citrus peel) with a probiotic bacterium capable of producing beneficial metabolites (e.g. PGPR *B. velezensis* AP203 and its nematicidal metabolites) may increase the efficacy of disease control in plant hosts, what is also referred to as a ‘synbiotic’ inoculum containing compatible prebiotic and probiotic agents.

The results from the greenhouse tests suggested that cell pellet, culture broth, and supernatant of *B. velezensis* AP203 with 1.0% (w/v) orange peel amended media significantly increased soybean and cotton plant growth (root length) compared to the *M. incognita* inoculated positive control. In addition, the numbers of *M. incognita* eggs of cell pellet, culture broth, and supernatant were reduced in the roots of cotton and soybean compared to the *M. incognita* inoculated positive control. However, there were no significant differences between cell pellet, culture broth, or supernatant of *B. velezensis* AP203 amended with orange peel. Previous studies reported that extracts of fresh orange peel significantly reduced *M. incognita* eggs and J2 in planta and in vitro (Faye, 2017; Raviv et al., 2005; Tsai, 2008). Recent studies also showed that *B. velezensis* strains were capable of enhancing cotton and soybean yields and reducing *M. incognita* egg populations in a greenhouse, micro plots, and field experiments (Xiang et al., 2017a, b). This is the first study to evaluate the combination of a PGPR *B. velezensis* strain and an orange peel amendment in antagonizing *M. incognita* populations. Agricultural waste can be an environmental problem, and waste management is an enormous challenge worldwide. The use of orange peel to promote the efficacy of PGPR-mediated plant pathogenic nematode control may reduce the need for chemical nematicides and improve plant and soil health. The present findings indicate the potential for *B. velezensis* AP203 and orange peel amendments to reduce *M. incognita* population density and increase yield under field conditions, thereby providing an alternative option to chemical nematicides. In addition to improving plant health and suppressing plant-parasitic nematodes, orange peel amendment to soils may also enhance soil nutrient levels. A recent study showed that agricultural waste orange peel significantly enhanced soil nutrient levels and regenerated tropical forest vegetation in Costa Rica (Treuer et al., 2018). Taken together, the results of this and other studies suggest that this synbiotic treatment may be a cost-effective strategy of benefit to sustainable agricultural practices.

The four metabolites that were specifically produced by *B. velezensis* AP203 when grown on an orange peel substrate may have direct inhibitory effects on *M. incognita* eggs and J2 viability. A previous study reported that the volatile organic compounds phenol, propyl benzene, propanone, and 1-ethenyl-4-methoxy benzene produced by *B. megaterium* YFM3.25 showed nematicidal effects and significantly reduced *M. incognita* eggs in pot experiments (Huang et al., 2010). Orange peel contains pectin, limonene, and phenolic compounds that have antioxidant properties and exerts beneficial effects on plant health (Bocco et al., 1998; Rafiq et al., 2018). The increased *M. incognita* mortality observed in this study may be due to a combination of metabolites producing by *B. velezensis* AP203 and derived from orange peel. These data support the conclusion that the metabolome of *B. velezensis* AP203 is significantly altered when this strain is grown on OPP, resulting in the expression of previously identified nematicidal compounds which were not observed when this strain was grown on glucose as a sole carbon source. No doubt, the chemical milieu of citrus peel includes a greater complexity of carbon sources and organic compounds, relative to growth on glucose alone, may elicit changes in *B. velezensis* gene expression. Further experiments should explore the changes in secondary metabolite expression for *B. velezensis* AP203 and other bioactive PGPR strains within the rhizosphere, as affected by the presence of pectin-rich amendments or other compatible prebiotic complex carbohydrates. This study is the first report that a *B. velezensis* strain in combination with an orange peel prebiotic amendment can inhibit the viability of *M. incognita* eggs and J2, mediated at least in part through the production of bioactive secondary metabolites. Thus, treatments that combine *B. velezensis* strains with nematicidal potential along with an orange peel amendment are predicted to more effectively reduce plant-pathogenic nematode population density in planta.

In conclusion, *B. velezensis* AP203 when combined with an orange peel amendment significantly reduced *M. incognita* populations in vitro and in planta. Hence, the combined use of *B. velezensis* strains and orange peel represents a promising and sustainable biological control technique for plant-parasitic nematodes.
